# Understanding Health Professional Responses to Service Disinvestment: A Qualitative Study

**DOI:** 10.15171/ijhpm.2019.20

**Published:** 2019-04-17

**Authors:** Deb Mitchell, Lisa O’Brien, Anne Bardoel, Terry Haines

**Affiliations:** ^1^ Monash Health Community, Monash Health, Dandenong, VIC, Australia.; ^2^ Occupational Therapy Department, School of Primary and Allied Health Care, Monash University, Frankston, VIC, Australia.; ^3^ Department of Management and Marketing, Swinburne University of Technology, Hawthorn, VIC, Australia.; ^4^ School of Primary and Allied Health Care, Monash University, Frankston, VIC, Australia.

**Keywords:** Disinvestment, Staff Reactions, Weekend Allied Health Services, Service Change, Healthcare

## Abstract

**Background:** Disinvestment from inefficient health services may be a potential solution to rising healthcare costs, but there has been poor uptake of disinvestment recommendations. This Australian study aims to understand how health professionals react when confronted with a plan to disinvest from a health service they previously provided to their patients.

**Methods:** This qualitative study took place prior to the disinvestment phase of a trial which removed weekend allied health services from acute hospital wards, to evaluate the effectiveness and cost effectiveness of the service. Observations and focus groups were used to collect data from 156 participants which was analysed thematically.

**Results:** Initial reactions to the disinvestment were almost universally negative, with staff extremely concerned about the impact on the safety and quality of patient care and planning ways to circumvent the trial. Removal of existing services was perceived as a loss and created a direct threat to some clinicians’ professional identity. With time, discussion, and understanding of the project’s context, some staff moved towards acceptance and perceived the trial as an opportunity, particularly given the service was to be reinstated after the disinvestment.

**Conclusion:** Clinicians and health service managers are protective of the services they deliver and can create barriers to disinvestment. Even when services are removed to ascertain their value, health professionals may continue to provide services to their patients. Measuring the impact of the disinvestment may assist staff to accept the removal of a service.

## Background

The cost of healthcare in Western countries is rising due to aging populations, increases in chronic disease, and higher expectations of consumers.^1^ Each year more techniques, procedures and medications are added to publicly funded health systems than are removed, contributing further to rising healthcare costs.^2^ Economists have questioned the sustainability of existing models and suggested disinvestment from some healthcare services.^3^ Disinvestment is defined as the complete or partial withdrawal of resources from healthcare services that are regarded as either unsafe, ineffective and/or inefficient, with those resources shifted to health services with greater evidence of clinical or cost-effectiveness.^4^

There appears to be a double standard in the burden of evidence to support new versus existing services. New treatments usually must prove themselves safe, effective and cost effective, yet there has been little appetite for applying the same standards to existing services.^2,5^ Institutions such as the National Institute for Health and Care Excellence in the United Kingdom have published “Do not do” lists of medical procedures that have little or no evidence of effectiveness in an attempt to drive disinvestment.^6^ However, the impact of this approach has been openly questioned given the poor uptake of recommendations from these campaigns.^6,7^ These failures have arisen despite many clinicians agreeing that pressured health service budgets should not be used to fund low value health services.^6^ The support of clinicians has been found to be critical in the implementation of disinvestment decisions,^8^ but those attempting disinvestment have found a number of barriers including health professionals’ responses to disinvestment initiatives.^10,11^

Healthcare staff must stop providing a service they are accustomed to providing for disinvestment to be successful. Implementing change in healthcare is acknowledged as challenging.^11,12^ For change to be successful, both the change in actions and the transition – what people feel, experience and perceive as important – must be understood and managed.^12^ Previous researchers have likened healthcare workers’ responses during significant workplace change to the reactions of grief observed by Kubler-Ross,^11-13^ after the death of a loved one. If these responses are anticipated, decision-makers may be able to provide support at each stage to assist staff to accept the change process.

This study took place at the start of a larger trial investigating the effectiveness of weekend allied health services on acute wards.^14^ Allied health services, such as dietetics and physiotherapy, are among those that are commonly provided on weekends in hospitals internationally,^15,16^ and are seen by those who manage them as improving patient flow and quality of care and reducing adverse incidents.^17^ Although there is evidence to support the provision of weekend allied health services in subacute wards,^18-20^ at the time of this study there was little evidence that these services were effective on acute medical and surgical wards.^14^ The larger trial proposed the removal of weekend allied health services for a period of 6 months, whilst measuring the impact on patient outcomes such as length of hospital stay, adverse outcomes such as falls and patient feedback. The staff on the impacted wards and those providing allied health services were accustomed to having weekend allied health services available. This study’s research question was: How do health practitioners react when confronted with a plan to disinvest from a health service that they previously provided to their patients?

## Methods

### Design

This qualitative study took place prior to and during the disinvestment phase of a multisite stepped wedge cluster randomised controlled trial, which removed the weekend allied health service from twelve acute medical and surgical wards at two Australian public hospitals.^14,21,22^ The aim of the larger study was to ascertain the value of weekend allied health services to assist in planning services in the future. After a period of no weekend allied health service, a new model of service was to be designed, using feedback from ward stakeholders, and implemented. The effectiveness of the previous model of weekend allied health service could then be compared to the new model and to no weekend service.^14^ Staff who previously provided weekend allied health services to these wards were redeployed to other areas of the health service. A qualitative approach with a pragmatic lens^23^ was adopted because we aimed to use the focus groups as part of the change management strategy – to allow staff to discuss their concerns as well as an opportunity to explain to paucity of evidence for weekend allied health services and the safety measures included in the larger trial. We also wanted to examine and understand staff behaviour prior to and during the change in service to assist decision-makers to understand staff responses to disinvestment research. Observations during staff meetings, project launches, and focus groups were used to record staff reactions to the proposal and early stages of the disinvestment project.

### Participants and Settings

Participants were healthcare workers from the 12 acute wards from two tertiary hospitals (denoted by Hospital A and Hospital B, see Table 1) in Melbourne, VIC, Australia. Focus groups were conducted with the multi-disciplinary team in each of the wards participating in the disinvestment trial. A total of 156 health workers from medical, allied health and nursing backgrounds participated in fifteen focus groups (Table 1).

**Table 1:** Focus Group Participants

	**Doctor**	**Nurse**	**Dietitian**	**Physio-** **therapist**	**Occupational Therapist**	**Speech Pathologist**	**Social Worker**	**Allied Health** **Assistant**
**Hospital A** Medical Focus Group	8							
Ward A – Focus group 1		3						
Ward A – Focus group 2			1	1	1	1	1	1
Ward B		7	1	1	1	1		
Ward C – Focus group 1		1	1	2	2	1	1	1
Ward C – Focus group 2		7						
Ward D		5	1	1			1	
Ward E		11	1	1	1			
Ward F		12	1	2	1	1	1	
**Hospital B**	
Ward A		6	2	1	1			
Ward B		4	1	1				
Ward C	2	8	1	5	2	2	1	
Ward D		4	1		1	2	1	
Ward E		6	1	2		2	1	
Ward F		6	1	4	1	2		
**Total**	**10**	**80**	**13**	**21**	**11**	**12**	**7**	**2**
							**Total**	**156**

### Description of the Disinvestment Project

**Figure 1:**
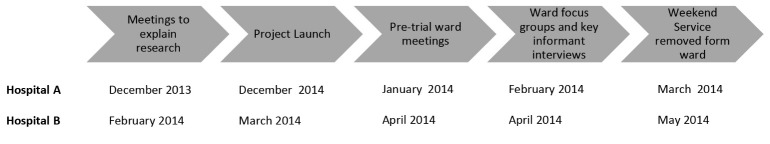
Communication and Data Collection Timeline.

The existing service at Hospital A comprised dietetics, physiotherapy, occupational therapy, speech pathology and social work, supported by allied health assistants. At Hospital B, the weekend allied health service included only physiotherapy and occupational therapy. The research team developed a communication strategy which involved explaining the larger study to staff at multiple time-points before and during the trial (see Figure 1). Meetings were held to explain the research idea, then once executive and ethics committee approval for the study was obtained at each site, the research team presented at management and senior clinical meetings, and at meetings on each ward, to introduce and explain the project.

### Procedure

Notes were taken by researchers (XX and XY) at each pre-research meeting, specifically noting staff questions, comments, and non-verbal reactions to the proposed disinvestment study. Focus groups with ward-based staff and the medical staff were held during routine ward meetings and staff education time, creating a convenience sample of the healthcare team working on the wards (see Table 1). The investigators developed a semi-structured interview schedule to guide these focus groups (see Table 2). The questions aimed to explore the participants’ views of the disinvestment research itself. The data collection took place prior to the withdrawal of the weekend allied health service, from December 2013 until June 2014 at Hospital A and from March until September 2014 at Hospital B. The data collection took place within a 6-month period, prior to weekend allied health services being withdrawn from the wards. A number of staff were interviewed or observed more than once, with other staff hearing about the planned disinvestment for the first time in the focus groups. Each focus group was facilitated by one researcher (XY), with another present to take notes (XX) and were recorded and transcribed verbatim by XX. All reported quotes are from the focus groups. Participants gave informed, written consent to participate in the study. Ethical approval for the study was obtained from each hospital’s ethics committee.

**Table 2:** Semi-structured Focus Group Questions

1	What are the current duties of performed by the weekend allied health staff? Why are they performing these roles?
2	Are there any situations where you may break the rules of the research?
3	What concerns do you have about the withdrawal of the weekend allied health service?
4	What is the reasoning behind these concerns? Are they based on evidence, experience or something else?
5	Do patient safety measures like stopping rules and clinical exceptions reduce your concerns?
6	How do you feel about the concept of removing a service to ascertain its value?

### Analysis

Recordings, transcripts, researcher field-notes, and staff meeting observation notes were loaded into NVivo software version 10 (QSR international). After each focus group, memos were recorded, and new questions or prompts added to further explore emerging ideas during subsequent data collection. XX coded the data using thematic analysis methods.^24^ After familiarisation with the data, initial codes were identified, and a coding matrix developed into which the data was assigned. The codes were then grouped into overarching categories, some of which focussed on how the reactions to the research changed over time. In moving between the data and categories, the data made sense in the light of researchers’ own experiences and other research about responses to change, and the categories could then be grouped into themes. Themes were defined as a group of views which reflected a participant’s preoccupation with an issue. Explanatory accounts of these themes were then developed to reflect the meaning of the original data and to seek wider application of these themes. Interpretations were discussed with other researchers (XY, YZ, and XZ) for clarity and trustworthiness, and disagreements were resolved by discussion. The analyses leading to the formation of themes were checked for bias by a researcher with a professional background in business management (YZ), as the other 3 researchers had professional backgrounds in allied health.

### Background, Training, and Preconceptions of Investigators

None of the investigators were currently working on weekends. XX, XY and XZ had previously worked on weekends on acute medical and surgical wards. XX and XZ were employed by one of the participating hospitals and were colleagues of the staff being interviewed at that site. All except one were also investigators on the larger study investigating the effectiveness and cost effectiveness of weekend allied health services in acute medical and surgical wards. Investigators anticipated that staff providing and referring to weekend allied health services might magnify their value to preserve the funding allocated to these services.

## Results

Analysis of the data revealed five themes. All appeared to be linked to a sense of control, or lack thereof, over the services provided to patients in their care. We observed a temporal aspect in the data, in that the nature of responses appeared to change over time, and so we present the themes in chronological order.

### Immediate Negative Reactions – Increased Risk and Reduced Patient Flow With Potential for Major Impact

A major theme in meetings to explain the research and focus groups was the concern that service disinvestment would cause an increased risk to patients and staff as many participants believed that weekend allied health services reduced these risks. Allied health and nursing staff anticipated an increased risk of patient falls, potentially resulting in harm to both patients and nurses if allied health were not there to get patients out of bed for the first time after surgery.

Some staff imagined unlikely worst-case scenarios, such as a bariatric patient being discharged from hospital to a nursing home instead of going home and a young man sustaining a brain injury due to a fall from a poorly chosen shower stool.

“*If a forty-year-old bariatric patient comes out of ICU [intensive care unit] and they’re deconditioned, allied health make a rehab[ilitation] plan and they get them going. If they’re [allied health] not there, they [the patient] just ends up in a nursing home!” [Nurse 2, Hospital B].*

Others described more everyday tasks they felt would be more dangerous without specialist allied health input, such as a speech pathologist assessing a patient’s ability to swallow after a stroke or assisting a patient for the first time out of bed post-surgery.

“*We might not choose the appropriate gait aid because we don’t have the necessary skills to know which is the appropriate gait aid to use. The risk is …. patients falling and nurses injuring themselves” [Nurse 1, Hospital A].*

Staff described the likely alternative of keeping heavy and/or very immobile patients in bed until allied health staff returned on Monday, to reduce the risk to staff. This prolonged bedrest was seen to potentially increase the risk of patient deterioration. Nursing staff were particularly concerned about not having a physiotherapist to assist with patient mobility.

“*If someone is a fairly difficult transfer and they have had surgery Friday, they get stuck in bed all weekend until the physio[therapist] sees them Monday. That’s two days when they’re more likely to have complications, because they haven’t been out of bed” [Nurse 1, Hospital B].*

### The Disinvestment Research Project Is an Excuse to Save Money

Allied health, medical, and nursing staff were observed in meetings and focus groups to voice the opinion that the project was cost cutting exercise and once the services were removed, they would not be replaced. Allied health staff predicted they would need to work longer (unpaid) hours on Fridays to prepare for the weekend by arranging equipment and services for people who may be discharged over the weekend. Monday mornings were already busy after a weekend with reduced allied health cover and many felt this would be exacerbated with no weekend service. Nurses felt their workloads would increase on weekends when there were already less non-nursing staff working, leaving them to pick up the slack.

“*On the weekend we’re down on pathology staff, so we’re doing pathology work, we’re down an RMO [resident medical officer], so we’re picking up their work. We’re absolutely skeleton staff” [Nurse 3, Hospital A].*

“*I think we have enough to do. I think we already have too much to do! I see nursing in a rush, I see cut corners everywhere. I see quality of patient care replaced with quantity, and I see this increasing. And I feel that fiercely” [Nurse 4, Hospital A].*

Allied health staff saw withdrawing the weekend service as a potential threat to the weekday services, predicting if allied health could be removed from wards on the weekend, the next step would be a push to remove it during the week. Several allied health staff voiced the threat they perceived to their role if they were not seen as required on the weekend.

*“Whether they [medical staff] see us as necessary in what we do or whether they see it as ‘well if you’re going to take it away, you don’t really need to be there anyway’”* [Allied Health 1, Hospital B].

These concerns were reduced when the research team explained that the weekend allied health staff were still employed but had been reallocated to other wards to measure the impact of the service there. Explanations of the project’s second stage, where weekend allied health services would be reintroduced, using a model designed with feedback from the wards, and the resources allocated to evaluating the service, appeared to be important in reducing concerns about perceived funding cuts.

### The Project Was Incompatible With the Values of the Health Service

A theme that persisted over time related to how disinvestment integrated with the values of the health service. Staff felt that disinvesting in weekend allied health services conflicted with hospital values and initiatives, such as those aimed at improving patient flow. In meetings and focus groups participants were observed and anticipated that disinvesting in weekend allied health services would lengthen inpatient stays and subsequently impede patient flow. Some staff warned of “bed block,” a situation where patients waiting for beds in the emergency department cannot be admitted because other patients have not been discharged. This was because patients were often referred to allied health staff to check they were “safe for discharge” and ward staff thought patients would not be discharged without allied health staff to check they were walking and transferring safely and had the equipment they needed to be safe at home. Staff were particularly concerned about patient flow because emergency departments had length of stay targets with financial consequences for the health service. Wards were under pressure to discharge patients to allow admissions from emergency.

*“If people are staying longer, they’ll take up a bed that someone else can’t go into, and that will delay someone in ED [the emergency department] the implication is bed blocking – potentially”* [Nurse 3, Hospital B].

Reductions in allied health staff on the weekends seemed contrary when patient flow coordinators asking the nurse in charge of the ward every morning which patients could be discharged to allow others to be admitted. One justification for having weekend allied health staff had been to facilitate discharge for patients who needed, for example, a gait aid to enable safe to be discharge from hospital. Some teams, such as the senior doctors at Hospital B, were increasing weekend staffing as part of a push toward a twenty-four-hour, seven days per week hospital service.

“*We [the medical consultants] have taken the view that we are running a 24/7 hospital so we’re now talking about whether we have consultants on at night. Which is going in exactly the opposite direction [to this study]. We actually do extra work on weekends to get them out!” [Doctor 1, Hospital B].*

Participants also perceived the restriction of patient access to weekend allied health, as incompatible with the health service’s value of delivering high quality, evidence-based care. Two areas of concern were the reduced access to speech pathologists to assess a patient’s ability to safely swallow and access to early intervention after stroke. These conditions were designated priorities for weekend allied health services as published clinical guidelines recommended provision of these services as part of best patient care. Staff also felt that the current weekend allied health service was restricted and represented the minimum needed and any less was providing care below the level they felt was acceptable.

*“For speech [pathologists] the big one is nil by mouth [fasting]. If they are not assessed [for swallowing safety], then they are nil by mouth for a couple of day; it sets them up for malnutrition, dehydration, [and] increased length of stay”* [Allied Health 2, Hospital A].

*“We have stroke patients that go onto the wards on a Friday afternoon. Now they won’t be seen until Monday. The stroke guidelines suggest that a patient who has had a stroke should be reviewed [by the allied health team] within 48 hours!”* [Allied Health 3, Hospital A].

Ward staff anticipated feeling embarrassed to tell patients and families that there was no allied health staff available on the weekend and so patients would need to wait until Monday to access the equipment they needed to go home. They felt that removing weekend allied health services represented an unacceptable quality of care and would impact on the reputation of the health service.

*“It also looks bad. If relatives come in here and we say, ‘Sorry we can’t feed your father until Monday because there’s no speech therapist’ that looks bad on the hospital. [Or] ‘We can’t get a physio[therapist] on the weekend.’ And also, things like not feeding patients, getting the right diet, because there’s no dietitian. To me, that’s what people remember”* [Nurse 2, Hospital A].

### Planning to Bend the Rules to Avoid the Project

A reaction observed at initial discussions about the project was staff planning ways of circumventing the trial protocol to continue to provide patients with weekend allied health services; a theme further explored in the focus groups. Medical staff at both hospitals discussed moving patients between wards to access weekend allied health services for their patients on the weekends. Allied health staff planned to work extra hours on Fridays and Mondays to reduce the impact of the study on patient care. Others considered providing weekend services on wards whose services had been withdrawn.

### Interviewer: Do you think you’d have any problems abiding by the rules of the research?

*“Yes I would - I know the nursing staff and I think they will ask me to do things like bring them a frame if they need it and I think I will do it. There are a lot of things we’re told we can’t do but we do them because it’s the safer option”* [Allied Health 3, Hospital A].

The focus groups and repeated meetings provided the research staff with forums to explain the potential longer-term consequences of breaking trial rules by providing services on wards where the impact of having no service was being measured. The forums were also a time where the participants could voice their concerns and questions to the researchers, who were there to listen and provide answers, as well as collect data.

### Disinvestment as Opportunity

Several staff perceived the concept of measuring the impact of a service as it was removed to be a positive aspect of the disinvestment model used. They acknowledged that while their initial responses had been negative, on reflection, they saw measuring the impact of the withdrawal of service on patient and hospital outcomes as a tool to evaluate the service being withdrawn. They perceived the lack of evidence for weekend allied health services to be a threat to its future funding and the opportunity of designing a new model of service as chance to improve the service in the future.

### Interviewer: What about the concept that a hospital could take away a service for the purpose of evaluating it - how do you feel about that concept?

*“It makes me feel a bit uncomfortable initially, except that we know that those services can get arbitrarily withdrawn as a budget decision in the absence of evidence, it’s better to do it in a controlled fashion .... Basically, what tends to happen in the past is that it gets withdrawn, we all suffer and complain, then it gets re-instated and we actually feel better, but we don’t know whether it is or isn’t better”* [Doctor 1, Hospital A].

*“I guess it’s surprising the lack of data that’s out there as well. It would be good to have some level of evidence to support allied health intervention on the weekend”* [Allied Health 2, Hospital B].

With the passage of time from the introduction of the disinvestment proposal, some staff had discussed and clarified their thoughts about the project and some appeared to be more comfortable with the idea of removing a service to ascertain its value. The reaction of allied health staff to “workarounds” to avoid the impact of the research changed and participants voiced concerns about the potential for people to influence the research results by not abiding by the research rules. Some appeared to move from wanting to continue to provide the service towards wanting staff to abide by the research rules so a true measure of the impact of the removing service in the longer term could be obtained.

*“What we’re concerned about is, if people see more patients on Friday because they haven’t got a weekend service, the outcome is not going to reflect that we need a weekend service, because we’re doing additional work on other days. Then at the end of the research they will say ‘let’s not give you a weekend service because you don’t need it’”* [Allied Health 3, Hospital B].

By voicing their support for aiding by the rule of the research in the focus groups and during everyday interactions with other staff, these participants became local advocates, or champions, of the project, and assisted the research team to explain the value of the disinvestment trial to other ward staff who viewed the project in negative light. A group of nursing staff also saw the project as an opportunity and expressed confidence in their ability to undertake tasks usually performed by allied health during the week and perceived this to be either part of their role or an opportunity to practice or expand on existing skills. This was particularly voiced by more experienced nurses, who had previously worked with less weekend allied health and who appreciated the opportunity to provide more holistic care for their patients.

*“On the weekend, we have the opportunity to be forced to manage certain types of patients. It makes us work more autonomously, without having to rely on – “the physio[therapist] will be here soon to manage the trache[ostomy] or get the patient out of bed.” It puts the onus back on the nursing staff which builds their skill level”* [Nurse 4, Hospital B].

*“Doctors want physio[therapist]s to do chest physio[therapy] and I say to them, ‘We do chest physio[therapy], saline nebulizers, we do active breathing cycles, what further do you want? That patient is coughing and able to expectorate, they are doing bubble PEP [positive expiratory pressure – a technique used to help clear lung secretions], do we need to have physio[therapy] on top of that? We can do that!’”* [Nurse 5, Hospital B].

## Discussion

**Figure 2:**
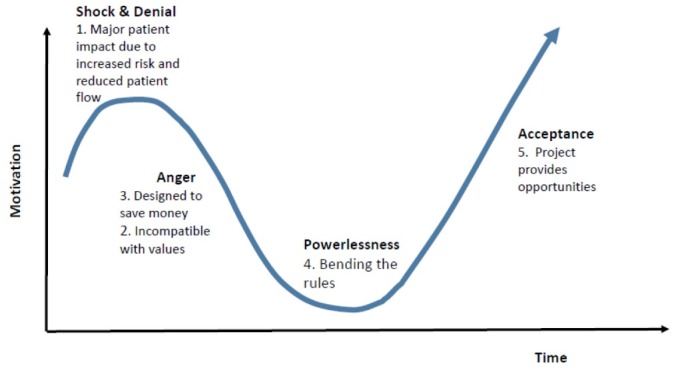
Staff Reactions to Disinvestment Mapped Onto Change Curve.^25^

We found five themes amongst staff reactions to the impending disinvestment and all reflected the lack of control the staff had over the services provided by the health service. Disinvestment in usual service represents a loss to both clinicians and patients, and so staff were reacting to that loss. These reactions followed a similar pattern to those first observed by Elisabeth Kubler-Ross, when studying emotional responses to the grief of losing a close relative.^25^ At the beginning, most participants were seen to express shock and denial, which then changed to stronger feelings such as anger. This was followed by a low point, described by Kubler-Ross as depression. After time, individuals begin to accept what has happened and integrate the reality of the situation into their identity and understanding of the self. Kubler-Ross found the pattern of emotions to be mostly consistent between people but noted differences in the depth of emotions and the time taken to move through the stage.^25^ She also noted people displayed similar responses to significant change and illustrated this using the Change Curve. We have mapped the response we observed in staff to the Change Curve (see Figure 2). We have made some assumptions in coming to the conclusion that there may be a linear progression through these stages. We observed individuals at different stages within the same focus group meetings in a cross-sectional sense. We did not observe how these same individuals changed their perspectives over time in a longitudinal sense. Therefore, it is possible that some did not experience all of the stages, some may have skipped stages, while others may not have progressed all the way along the curve to acceptance.

There were a near-universal negative responses observed from participants when they first heard of the plan to disinvest, with some staff predicting dire outcomes, despite the short-term nature of the disinvestment. Staff perceived that the removal of service would cause severe restrictions to patient flow, leaving patients in the emergency department waiting for beds and that patient and staff injuries would increase. Some warned of catastrophic outcomes such as severe patient injuries and even deaths. We observed anger as staff perceived the research project was part of a longer-term plan to disinvest in weekend allied health service, which they saw as incompatible with hospital values of providing high quality care. Plans to bend the research rules were a type of sabotage as staff tried to find ways around the project and may have reflected feelings of powerlessness, rather than “depression” observed by Kubler-Ross.^25^ Clinicians from each profession, perhaps reflecting past experience of funding “cuts,” described ways to work around to rules of the research to continue to provide allied health services on the weekends. With time, some participants recognised the larger research study as an opportunity to build evidence for weekend services (allied health) or to provide holistic care (nurses), and then acted as local champions to assist other to accept the project.

The Kubler-Ross Change Curve has been used to explain strong early resistance to change and the modification of staff responses over time in other research, such as enforced changes in technology used in intensive care,^26^ implementation of an electronic medical record^11^ and when executing multidisciplinary teams in general practice.^13^ All studies emphasised the time needed for this resistance to recede, despite communication and feedback strategies. Over time, participants in our study became more open to the idea of the study once they understood the context of the disinvestment; weekend services on acute medical and surgical wards had poor evidence to support their provision^21^ and this study would provide that evidence if the service affected patient outcomes.

The reported experience of staff did not, however, always uniformly follow the same pattern. Some staff accepted the project with minimal resistance. Even within health professional disciplines on the same ward, there were contrasting reactions. For example, some nurses reacted to the prospect of losing their weekend allied health service by complaining that their own workload would be increased or that they would be asked to do things outside of their skill set. There were undertones of disempowerment in these expressions. However, others expressed a strengthening of their professional identity in the roles they had performed prior to allied health presence on weekends.

The theoretical background on professional identity may assist in understanding the observed resistance to disinvestment in the weekend allied health service.^27^ Staff saw the project as a threat to patient safety and quality of care and to meeting patient flow targets, resulting in conflict between the healthcare workers’ professional and organisational identities.^28^ Similar conflict between professional and organisational identity have been observed in research involving nursing and medical staff. Nurses have been found to put their “nursing ideals” into practice even when this meant going against the rules of the organisation they worked for.^28^ Whilst doctors tend to be willing to adhere to recommendations made by a physician in charge about administrative duties, they felt that complying with advice on clinical practice was optional. Following an order without independently evaluating it was seen to be equivalent to being irresponsible professionally.^29^

Participants who, in later forums, highlighted the lack of evidence for the service and the unique opportunity of measuring its impact appeared to have moved toward acceptance, recognising that the project was going ahead regardless and recognising potential benefits. They appeared to have gone through a transition from focussing on the risks of the project (which may have threatened their professional role in keeping patients safe) to seeing the opportunity of finding out if the weekend allied health service was efficient and effective (reinforcing their professional identity as evidence-based practitioners). They thus become “influential supporters” of the disinvestment project, a factor found to be vital in giving disinvestment programs legitimacy.^8^

Understanding initial staff reactions to loss and the time required for staff to accept change and grapple with the threats to professional identity may enable identification of strategies useful in facilitating disinvestment.^30,31^ When participants are actively resisting the change, they are likely to be firmly attached to the “old.”^31^ It may be possible to reduce fear and mistrust by explaining the safety mechanisms included in the project, such as the assessment and approval by an ethics committee and the existence of trial stopping rules.^14,21^ The research design included repeated forums in which staff could voice their concerns and researchers could answer or counter any arguments and this assisted staff to work through the stages of change. Open discussion of the teams’ concerns may also assist staff to explore new ways of thinking. By encouraging the discussion of ideas and listening to feedback regarding the project, researchers may increase participants’ attention to safety features of the research design. For example, several staff in this study were unaware of the scarcity of evidence for weekend allied health services before the project began. Reactions to change will be different for different groups and individuals^30^ and researchers need to be aware that some may remain angry and attempt to sabotage the project. Highlighting the potential impact of trial sabotage on project results and the subsequent resourcing implications may discourage these plans.

Disinvestment in currently provided health services can result in a clash between those professionals advocating the change and those who see the disinvestment as a threat to their ability to provide good patient care.^17,19^ The disinvestment may be more likely to be accepted by staff when they are provided with evidence of better (or not worsened) patient outcomes.^8,31^ The presence of consumers in the decision-making process^10,31^ may also assist staff in accepting that limiting resources is acceptable. However, the presence of consumers may be challenging with consumer protests resulting in failed attempts at disinvestment.^9^

There are several strengths and limitations of this study that should be considered. First, this research was conducted within a single topic context area and across only two health services. This limits the generalisability of our findings and conclusions regarding how staff react to disinvestment and the likelihood that similar patterns would be observed in different contexts and settings. Second, the disinvestment in the present study was concurrently accompanied by a randomised controlled study seeking to evaluate the effective and cost effectiveness of the service being disinvested in.^18^ Therefore, it may be difficult to extrapolate the finding of this research to other disinvestment situations that did not include this rigorous evaluation component. This is because the presence of the data generation may have kept a number of staff “on side” with this disinvestment process under the belief that it would generate the evidence that would be used to protect the service for decades to come. Similarly, the evaluation may have changed the propensity for staff to use workarounds to avoid the disinvestment. As a change management strategy, the investigators took advantage of the randomised controlled study component of this process to highlight to staff that using workarounds would only serve to diminish the apparent effect of the intervention (weekend allied health) and subsequently create a case for its permanent removal. Understanding the impact of simultaneously conducting a randomised controlled trial along with the disinvestment, compared to just undertaking the dis alone, would require a comparator group to be generated that just sought to disinvest from the weekend allied health service and be kept blinded to the randomised controlled trial.

We examined the reactions to the removal of the weekend allied health service from the perspective of the multidisciplinary team. One hundred and fifty-six staff from eight professions were interviewed, all of whom worked on acute medical and surgical wards with weekend allied health services. The data were gathered and analysed by a team of experienced researchers, mainly allied health professionals, some of whom worked at one of the hospitals studied, as part of the change process for the larger study. This may have affected the collection and interpretation of data, as some staff may have been reluctant to voice concerns to their colleagues. To counteract this, staff employed by the university led the focus groups. As most of the research team had backgrounds in allied health, a researcher external to both the health services and allied health was included in the research team to assist with development of the data collection approach and interpretation. Personal biases, such as perceiving that staff working around restrictions was normal practice, were discussed between the principal investigator and the external researcher and strategies to limit their potential impact developed and implemented. The context of the disinvestment initiative may have led to different participant reactions to those to a disinvestment where no research was concurrently being done. This would have a direct impact on theme 5, particularly for the allied health participants who saw the project as an opportunity to build the evidence for the service. The evaluation paradigm employed should potentially be used in future disinvestments as it was an important factor in gaining staff acceptance. When this is not possible, reactions could potentially be more extreme, and emphasis should be placed on the evidence used to justify the disinvestment decision.

## Conclusion

Whilst new healthcare services need to show they are effective and cost-efficient to attract funding, those providing existing services face no such barriers. This study helps explain why the apparent double standard in the burden of evidence between new and existing health services goes unchallenged.^2,5^ Removal of existing services appears to create a direct threat to clinicians’ professional identity and precipitates an experience of loss for those healthcare professionals affected. This can become a strong disincentive for clinicians to organically question their own practice and self-initiate disinvestment from existing services, even if these services deliver marginal benefit, through overuse, misuse or waste.^33^ Bringing about changes through disinvestment is an important step in improving in healthcare services. But to reap the benefits healthcare workers need also to adapt and change accordingly. While some find it easy to move along the journey, but others find the individual transformations traumatic.

## Acknowledgements

The authors would also like to acknowledge the steering committee of the larger trial: Kelly-Ann Bowles, Tim Chiu, Marcelle Ghaly, Romi Haas, David Lescai, Kerry May, Donna Markham, Fiona McDermott, Kathleen Philip, Samantha Plumb, Mitchell Sarkies, Melina Shackell, and Elizabeth Skinner.

## Ethical issues

The study was approved by Monash Health Human Research Ethics Committee and Melbourne Health Human Research Ethics Committee, Australia.

## Competing interests

Two of the authors were colleagues of the study participants at one hospital and three of the authors have similar professional backgrounds to the study participants. This could potentially be perceived as a conflict of interest. None of the investigators managed or worked in weekend allied health services, reducing the potential for conflict of interest. Investigators anticipated that staff providing and referring to weekend allied health services may magnify their value in order to preserve the funding allocated to these services. This was mitigated in the larger study by reallocating funding allied health services in other areas of the hospital. A fourth investigator with a business management background was asked to check the manuscript for bias.

## Funding

Financial support for this study was provided in part by a grant from the National Health and Medical Research Council (NHMRC) Australia (ID 1060696) and from the Department of Health and Human Services, VIC, Australia. TH is supported by a Career Development Fellowship from the NHMRC (ID 169758). The funding agreements ensured the authors’ independence in designing the study, interpreting the data, writing, and publishing the report.

## Authors’ contributions

DM and LOB collected the data. DM performed initial analysis and drafted the article. All authors discussed and reviewed themes and their application to the wider field. All authors contributed to, reviewed, provided critical input into and gave approval to the final manuscript.

## Authors’ affiliations

^1^Monash Health Community, Monash Health, Dandenong, VIC, Australia. ^3^Occupational Therapy Department, School of Primary and Allied Health Care, Monash University, Frankston, VIC, Australia. ^3^Department of Management and Marketing, Swinburne University of Technology, Hawthorn, VIC, Australia. ^4^School of Primary and Allied Health Care, Monash University, Frankston, VIC, Australia.

## Key messages

**Implications for policy makers**

- Disinvestment from inefficient services may contribute toward managing the rising cost of healthcare and allow resources to be reallocated toward higher value care.
- Staff perceive disinvestment from services they routinely provide as a loss and so may resist the change.
- Disinvestment may threaten the professional identity of some staff impacted, resulting in negative reactions that may derail the disinvestment.
- Time, discussion and understanding of the context of the project may assist staff to move towards acceptance.

**Implications for public**

Some routinely provided healthcare services may not be the best use of limited resources. Healthcare staff are important decision makers in the provision of care and can influence whether or not changes to usual care are implemented. When services are stopped, the staff providing them experience loss and may resist the change. We studied the reactions of healthcare staff to a project that removed weekend allied health service (dietetics, occupational therapy, physiotherapy, social work and speech pathology) for acute, medical and surgical wards. Staff initially resisted the change, however with time, discussion, and increased understanding of the context of the project some staff moved towards acceptance.
